# 4-*tert*-Butyl-2-(4-*tert*-butyl­pyridin-2-yl)pyridinium nitrate

**DOI:** 10.1107/S1600536811028030

**Published:** 2011-07-16

**Authors:** Wen-Sheng Wu

**Affiliations:** a School of Chemistry and Chemical Engineering, Zhaoqing University, Zhaoqing 526061, People’s Republic of China

## Abstract

In the title compound, C_18_H_25_N_2_^+^·NO_3_^−^, the dihedral angle between the pyridine rings is 19.06 (10)°. In the crystal, the ions are linked into a three-dimensional network by N—H⋯O and C—H⋯O hydrogen-bonding inter­actions.

## Related literature

For background to the coordination chemistry and applications of bipyridine and its derivatives, see: Duan *et al.* (2010 ▶); Morrow & Trogler (1989 ▶); Noro *et al.* (2000 ▶); Yaghi *et al.* (1998 ▶); Huertas *et al.* (2001 ▶); Qin *et al.* (2002 ▶).
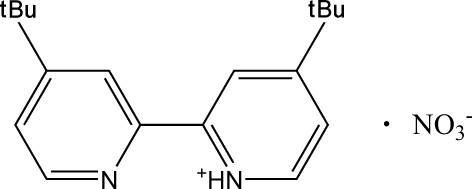


## Experimental

### 

#### Crystal data

C_18_H_25_N_2_^+^·NO_3_^−^
*M* *_r_* = 331.41
Orthorhombic, 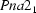

*a* = 11.606 (5) Å
*b* = 9.770 (4) Å
*c* = 16.199 (7) Å
*V* = 1836.8 (13) Å^3^
*Z* = 4
Mo *K*α radiation
μ = 0.08 mm^−1^
*T* = 273 K
0.29 × 0.24 × 0.19 mm

#### Data collection

Bruker SMART CCD area-detector diffractometer
Absorption correction: multi-scan (*SADABS*; Bruker, 2005 ▶) *T*_min_ = 0.962, *T*_max_ = 0.978
11773 measured reflections
1705 independent reflections
1314 reflections with *I* > 2σ(*I*)
*R*_int_ = 0.042

#### Refinement

*R*[*F* ^2^ > 2σ(*F* ^2^)] = 0.040
*wR*(*F* ^2^) = 0.112
*S* = 1.06
1705 reflections
227 parameters
1 restraint
H atoms treated by a mixture of independent and constrained refinement
Δρ_max_ = 0.14 e Å^−3^
Δρ_min_ = −0.13 e Å^−3^

### 

Data collection: *SMART* (Bruker, 2002 ▶); cell refinement: *SAINT* (Bruker, 2002 ▶); data reduction: *SAINT*; program(s) used to solve structure: *SHELXS97* (Sheldrick, 2008 ▶); program(s) used to refine structure: *SHELXL97* (Sheldrick, 2008 ▶); molecular graphics: *ORTEP-3 for Windows* (Farrugia, 1997 ▶); software used to prepare material for publication: *WinGX* (Farrugia, 1999 ▶).

## Supplementary Material

Crystal structure: contains datablock(s) global, I. DOI: 10.1107/S1600536811028030/rz2623sup1.cif

Structure factors: contains datablock(s) I. DOI: 10.1107/S1600536811028030/rz2623Isup2.hkl

Supplementary material file. DOI: 10.1107/S1600536811028030/rz2623Isup3.cml

Additional supplementary materials:  crystallographic information; 3D view; checkCIF report

## Figures and Tables

**Table 1:** Hydrogen-bond geometry (Å, °)

*D*—H⋯*A*	*D*—H	H⋯*A*	*D*⋯*A*	*D*—H⋯*A*
N1—H1*N*⋯O1^i^	1.00 (3)	1.89 (3)	2.716 (4)	137 (3)
C4—H4⋯O3^ii^	0.93	2.58	3.480 (4)	164
C7—H7⋯O3^ii^	0.93	2.49	3.389 (4)	163
C9—H9⋯O3^iii^	0.93	2.60	3.385 (4)	143

Symmetry codes: (i) 
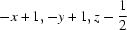
; (ii) 
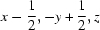
; (iii) 
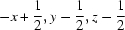
.

**Fig. 1.**
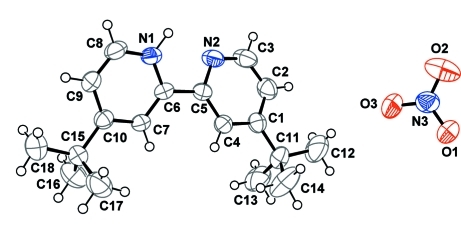
The molecular structure of title compound with displacement ellipsoids drawn at the 50% probability level.

## supplementary crystallographic information

### Comment

Metal complexes of bipyridine and its derivatives have been extensively studied because of their potential applications in catalysis (Morrow & Trogler, 1989; Noro *et al.*, 2000) and visible light driven water oxidation (Duan *et al.*, 2010). One of these compounds, 4,4'-di-*tert*-butyl-2,2'-bipyridine, has recently been used as ligand in coordination chemistry (Huertas *et al.*, 2001; Qin *et al.*, 2002). As a contribution to this research field, the crystal structure of the title complex containing a bipyridyl ligand is reported herein.

The asymmetric unit of the title compound (Fig. 1) consists of one 4-*tert*-butyl-2-(4-*tert*-butylpyridin-2-yl)pyridinium cation and one nitrate anion. In the cation, the dihedral angle between the planes of two pyridine rings is 19.06 (10)°. In the crystal, cations and anions are linked into a three-dimensional network by N—H···O and C—H···O hydrogen bonds (Table 1).

### Experimental

4,4'-Di-*tert*-butyl-2,2'-bipyridine (0.15 g, 0.56 mmol) and nitric acid (30%, 50 ml) were stirred for 20 min at 313 K.The solution was then filtered and left to evaporate slowly at room temperature. After three weeks, colourless laths and prisms of the title compound were isolated.

### Refinement

The H1N atom was located in a difference Fourier map and refined freely. All othe H atoms were placed in calculated positions and refined as riding, with C—H = 0.93–0.96 Å and *U*_iso_(H) = 1.2 *U*_eq_(C) or 1.5 *U*_eq_(C) for methyl H atoms. 1456 Friedel pairs were merged.

### Crystal data

**Table:**

C_18_H_25_N_2_^+^·NO_3_^−^	*F*(000) = 712.0
*M**_r_* = 331.41	*D*_x_ = 1.198 Mg m^−^^3^
Orthorhombic, *P**n**a*2_1_	Mo *K*α radiation, λ = 0.71073 Å
Hall symbol: P 2c -2n	Cell parameters from 1686 reflections
*a* = 11.606 (5) Å	θ = 2.4–25.1°
*b* = 9.770 (4) Å	µ = 0.08 mm^−^^1^
*c* = 16.199 (7) Å	*T* = 273 K
*V* = 1836.8 (13) Å^3^	Block, colourless
*Z* = 4	0.29 × 0.24 × 0.19 mm

### Data collection

**Table:**

Bruker SMART CCD area-detector diffractometer	1705 independent reflections
Radiation source: fine-focus sealed tube	1314 reflections with *I* > 2σ(*I*)
graphite	*R*_int_ = 0.042
φ and ω scans	θ_max_ = 25.2°, θ_min_ = 2.4°
Absorption correction: multi-scan (*SADABS*; Bruker, 2005)	*h* = −13→13
*T*_min_ = 0.962, *T*_max_ = 0.978	*k* = −11→11
11773 measured reflections	*l* = −18→19

### Refinement

**Table:**

Refinement on *F*^2^	Primary atom site location: structure-invariant direct methods
Least-squares matrix: full	Secondary atom site location: difference Fourier map
*R*[*F*^2^ > 2σ(*F*^2^)] = 0.040	Hydrogen site location: inferred from neighbouring sites
*wR*(*F*^2^) = 0.112	H atoms treated by a mixture of independent and constrained refinement
*S* = 1.06	*w* = 1/[σ^2^(*F*_o_^2^) + (0.064*P*)^2^ + 0.0558*P*] where *P* = (*F*_o_^2^ + 2*F*_c_^2^)/3
1705 reflections	(Δ/σ)_max_ < 0.001
227 parameters	Δρ_max_ = 0.14 e Å^−^^3^
1 restraint	Δρ_min_ = −0.13 e Å^−^^3^

### Special details

**Table:**

Geometry. All e.s.d.'s (except the e.s.d. in the dihedral angle between two l.s. planes) are estimated using the full covariance matrix. The cell e.s.d.'s are taken into account individually in the estimation of e.s.d.'s in distances, angles and torsion angles; correlations between e.s.d.'s in cell parameters are only used when they are defined by crystal symmetry. An approximate (isotropic) treatment of cell e.s.d.'s is used for estimating e.s.d.'s involving l.s. planes.
Refinement. Refinement of *F*^2^ against ALL reflections. The weighted *R*-factor *wR* and goodness of fit *S* are based on *F*^2^, conventional *R*-factors *R* are based on *F*, with *F* set to zero for negative *F*^2^. The threshold expression of *F*^2^ > σ(*F*^2^) is used only for calculating *R*-factors(gt) *etc*. and is not relevant to the choice of reflections for refinement. *R*-factors based on *F*^2^ are statistically about twice as large as those based on *F*, and *R*- factors based on ALL data will be even larger.

### Fractional atomic coordinates and isotropic or equivalent isotropic displacement parameters (Å^2^)

**Table:**

	*x*	*y*	*z*	*U*_iso_*/*U*_eq_	
O1	0.6857 (3)	0.6853 (3)	1.06496 (18)	0.0996 (11)	
O3	0.6436 (2)	0.5517 (3)	0.96612 (17)	0.0845 (9)	
O2	0.7322 (3)	0.7406 (4)	0.9424 (2)	0.1063 (11)	
N1	0.2126 (2)	0.0984 (3)	0.64095 (16)	0.0490 (7)	
H1N	0.280 (3)	0.143 (3)	0.613 (2)	0.059 (9)*	
N2	0.4248 (2)	0.1079 (3)	0.70159 (18)	0.0570 (8)	
N3	0.6866 (2)	0.6606 (3)	0.9902 (2)	0.0628 (8)	
C1	0.4700 (3)	0.1084 (3)	0.8731 (2)	0.0484 (8)	
C2	0.5555 (3)	0.1325 (4)	0.8155 (2)	0.0626 (10)	
H2	0.6306	0.1493	0.8327	0.075*	
C3	0.5294 (3)	0.1316 (5)	0.7323 (3)	0.0669 (10)	
H3	0.5888	0.1486	0.6952	0.080*	
C4	0.3599 (3)	0.0832 (3)	0.8416 (2)	0.0455 (7)	
H4	0.2988	0.0660	0.8773	0.055*	
C5	0.3418 (3)	0.0838 (3)	0.7571 (2)	0.0438 (7)	
C6	0.2263 (3)	0.0558 (3)	0.71989 (17)	0.0424 (7)	
C7	0.1355 (2)	−0.0087 (3)	0.7586 (2)	0.0438 (7)	
H7	0.1440	−0.0385	0.8128	0.053*	
C8	0.1142 (3)	0.0787 (4)	0.5994 (2)	0.0569 (9)	
H8	0.1078	0.1091	0.5452	0.068*	
C9	0.0233 (3)	0.0144 (3)	0.6360 (2)	0.0530 (8)	
H9	−0.0443	0.0001	0.6064	0.064*	
C10	0.0312 (3)	−0.0301 (3)	0.71786 (19)	0.0458 (8)	
C11	0.4935 (3)	0.1032 (4)	0.9658 (2)	0.0592 (9)	
C12	0.6147 (4)	0.1546 (7)	0.9880 (3)	0.119 (2)	
H12A	0.6713	0.0967	0.9625	0.178*	
H12B	0.6245	0.1524	1.0468	0.178*	
H12C	0.6241	0.2467	0.9685	0.178*	
C13	0.4872 (5)	−0.0481 (5)	0.9912 (3)	0.1001 (15)	
H13A	0.4117	−0.0831	0.9794	0.150*	
H13B	0.5025	−0.0562	1.0492	0.150*	
H13C	0.5436	−0.0994	0.9608	0.150*	
C14	0.4034 (5)	0.1836 (6)	1.0136 (3)	0.1090 (18)	
H14A	0.4091	0.2788	0.9996	0.163*	
H14B	0.4163	0.1721	1.0717	0.163*	
H14C	0.3280	0.1505	0.9996	0.163*	
C15	−0.0693 (3)	−0.0977 (3)	0.7623 (2)	0.0533 (8)	
C16	−0.0320 (4)	−0.2367 (4)	0.7949 (3)	0.0861 (13)	
H16A	−0.0095	−0.2941	0.7496	0.129*	
H16B	−0.0950	−0.2785	0.8239	0.129*	
H16C	0.0319	−0.2254	0.8318	0.129*	
C17	−0.1034 (4)	−0.0060 (4)	0.8356 (3)	0.0831 (13)	
H17A	−0.0393	0.0027	0.8727	0.125*	
H17B	−0.1674	−0.0463	0.8643	0.125*	
H17C	−0.1249	0.0829	0.8156	0.125*	
C18	−0.1747 (4)	−0.1165 (6)	0.7074 (4)	0.1001 (17)	
H18A	−0.1979	−0.0294	0.6855	0.150*	
H18B	−0.2366	−0.1547	0.7392	0.150*	
H18C	−0.1561	−0.1772	0.6627	0.150*	

### Atomic displacement parameters (Å^2^)

**Table:**

	*U*^11^	*U*^22^	*U*^33^	*U*^12^	*U*^13^	*U*^23^
O1	0.136 (3)	0.107 (2)	0.0558 (18)	−0.0491 (19)	0.0177 (17)	−0.0289 (16)
O3	0.097 (2)	0.094 (2)	0.0625 (18)	−0.0245 (17)	0.0054 (16)	−0.0279 (16)
O2	0.092 (2)	0.118 (2)	0.109 (3)	−0.0007 (19)	0.033 (2)	0.042 (2)
N1	0.0528 (17)	0.0565 (16)	0.0379 (15)	−0.0017 (12)	0.0009 (13)	0.0075 (12)
N2	0.0457 (16)	0.075 (2)	0.0502 (17)	−0.0018 (14)	0.0053 (14)	0.0172 (14)
N3	0.0545 (17)	0.074 (2)	0.060 (2)	0.0000 (15)	0.0104 (15)	−0.0011 (18)
C1	0.048 (2)	0.0409 (17)	0.056 (2)	−0.0002 (13)	−0.0028 (16)	0.0012 (14)
C2	0.046 (2)	0.071 (2)	0.071 (3)	−0.0058 (16)	−0.0048 (19)	0.0067 (18)
C3	0.052 (2)	0.085 (3)	0.064 (3)	−0.0027 (18)	0.0098 (19)	0.0224 (19)
C4	0.0428 (17)	0.0480 (17)	0.0458 (19)	−0.0006 (13)	0.0009 (14)	0.0000 (13)
C5	0.0462 (17)	0.0414 (16)	0.0437 (18)	0.0007 (12)	0.0028 (14)	0.0045 (13)
C6	0.0483 (18)	0.0458 (15)	0.0332 (17)	0.0039 (13)	0.0003 (13)	0.0019 (13)
C7	0.0480 (17)	0.0488 (16)	0.0344 (15)	−0.0007 (14)	−0.0008 (15)	0.0043 (13)
C8	0.071 (2)	0.062 (2)	0.0369 (18)	0.0006 (18)	−0.0048 (17)	0.0062 (15)
C9	0.0532 (19)	0.0607 (19)	0.0450 (18)	−0.0029 (15)	−0.0106 (16)	0.0019 (16)
C10	0.0502 (19)	0.0437 (17)	0.0434 (19)	0.0016 (13)	−0.0011 (14)	−0.0014 (14)
C11	0.056 (2)	0.067 (2)	0.055 (2)	0.0018 (16)	−0.0107 (17)	−0.0088 (18)
C12	0.086 (3)	0.193 (6)	0.077 (3)	−0.036 (4)	−0.022 (3)	−0.022 (4)
C13	0.131 (4)	0.106 (4)	0.063 (3)	0.003 (3)	−0.032 (3)	0.015 (3)
C14	0.112 (4)	0.147 (5)	0.068 (3)	0.044 (3)	−0.019 (3)	−0.039 (3)
C15	0.0503 (19)	0.058 (2)	0.052 (2)	−0.0104 (15)	0.0007 (17)	0.0003 (16)
C16	0.089 (3)	0.061 (2)	0.109 (3)	−0.014 (2)	0.012 (3)	0.017 (2)
C17	0.072 (3)	0.084 (3)	0.093 (3)	−0.014 (2)	0.025 (2)	−0.011 (2)
C18	0.072 (3)	0.140 (5)	0.088 (3)	−0.037 (3)	−0.014 (3)	0.006 (3)

### Geometric parameters (Å, °)

**Table:**

O1—N3	1.235 (4)	C11—C14	1.520 (6)
O3—N3	1.239 (4)	C11—C13	1.536 (6)
O2—N3	1.221 (4)	C11—C12	1.536 (6)
N1—C8	1.340 (4)	C12—H12A	0.9600
N1—C6	1.354 (4)	C12—H12B	0.9600
N1—H1N	1.00 (4)	C12—H12C	0.9600
N2—C3	1.332 (5)	C13—H13A	0.9600
N2—C5	1.339 (4)	C13—H13B	0.9600
C1—C2	1.383 (5)	C13—H13C	0.9600
C1—C4	1.398 (4)	C14—H14A	0.9600
C1—C11	1.527 (5)	C14—H14B	0.9600
C2—C3	1.382 (6)	C14—H14C	0.9600
C2—H2	0.9300	C15—C16	1.519 (5)
C3—H3	0.9300	C15—C18	1.524 (5)
C4—C5	1.384 (5)	C15—C17	1.540 (6)
C4—H4	0.9300	C16—H16A	0.9600
C5—C6	1.495 (4)	C16—H16B	0.9600
C6—C7	1.378 (4)	C16—H16C	0.9600
C7—C10	1.395 (4)	C17—H17A	0.9600
C7—H7	0.9300	C17—H17B	0.9600
C8—C9	1.364 (5)	C17—H17C	0.9600
C8—H8	0.9300	C18—H18A	0.9600
C9—C10	1.398 (4)	C18—H18B	0.9600
C9—H9	0.9300	C18—H18C	0.9600
C10—C15	1.521 (5)		
			
C8—N1—C6	122.0 (3)	C11—C12—H12A	109.5
C8—N1—H1N	119 (2)	C11—C12—H12B	109.5
C6—N1—H1N	118 (2)	H12A—C12—H12B	109.5
C3—N2—C5	115.8 (3)	C11—C12—H12C	109.5
O2—N3—O1	120.1 (4)	H12A—C12—H12C	109.5
O2—N3—O3	121.6 (4)	H12B—C12—H12C	109.5
O1—N3—O3	118.3 (3)	C11—C13—H13A	109.5
C2—C1—C4	116.1 (3)	C11—C13—H13B	109.5
C2—C1—C11	122.8 (3)	H13A—C13—H13B	109.5
C4—C1—C11	121.1 (3)	C11—C13—H13C	109.5
C3—C2—C1	120.0 (3)	H13A—C13—H13C	109.5
C3—C2—H2	120.0	H13B—C13—H13C	109.5
C1—C2—H2	120.0	C11—C14—H14A	109.5
N2—C3—C2	124.4 (3)	C11—C14—H14B	109.5
N2—C3—H3	117.8	H14A—C14—H14B	109.5
C2—C3—H3	117.8	C11—C14—H14C	109.5
C5—C4—C1	119.9 (3)	H14A—C14—H14C	109.5
C5—C4—H4	120.0	H14B—C14—H14C	109.5
C1—C4—H4	120.0	C16—C15—C10	109.6 (3)
N2—C5—C4	123.7 (3)	C16—C15—C18	108.9 (3)
N2—C5—C6	114.0 (3)	C10—C15—C18	113.0 (3)
C4—C5—C6	122.3 (3)	C16—C15—C17	109.0 (4)
N1—C6—C7	118.7 (3)	C10—C15—C17	108.0 (3)
N1—C6—C5	115.5 (3)	C18—C15—C17	108.3 (3)
C7—C6—C5	125.9 (3)	C15—C16—H16A	109.5
C6—C7—C10	121.1 (3)	C15—C16—H16B	109.5
C6—C7—H7	119.4	H16A—C16—H16B	109.5
C10—C7—H7	119.4	C15—C16—H16C	109.5
N1—C8—C9	120.5 (3)	H16A—C16—H16C	109.5
N1—C8—H8	119.8	H16B—C16—H16C	109.5
C9—C8—H8	119.8	C15—C17—H17A	109.5
C8—C9—C10	120.3 (3)	C15—C17—H17B	109.5
C8—C9—H9	119.8	H17A—C17—H17B	109.5
C10—C9—H9	119.8	C15—C17—H17C	109.5
C7—C10—C9	117.3 (3)	H17A—C17—H17C	109.5
C7—C10—C15	120.4 (3)	H17B—C17—H17C	109.5
C9—C10—C15	122.3 (3)	C15—C18—H18A	109.5
C14—C11—C1	111.2 (3)	C15—C18—H18B	109.5
C14—C11—C13	109.1 (4)	H18A—C18—H18B	109.5
C1—C11—C13	106.7 (3)	C15—C18—H18C	109.5
C14—C11—C12	110.0 (4)	H18A—C18—H18C	109.5
C1—C11—C12	112.5 (3)	H18B—C18—H18C	109.5
C13—C11—C12	107.2 (4)		
			
C4—C1—C2—C3	0.4 (5)	C6—N1—C8—C9	0.2 (5)
C11—C1—C2—C3	177.9 (4)	N1—C8—C9—C10	0.9 (5)
C5—N2—C3—C2	−0.1 (6)	C6—C7—C10—C9	1.1 (4)
C1—C2—C3—N2	−0.3 (7)	C6—C7—C10—C15	−178.2 (3)
C2—C1—C4—C5	−0.2 (4)	C8—C9—C10—C7	−1.5 (5)
C11—C1—C4—C5	−177.7 (3)	C8—C9—C10—C15	177.8 (3)
C3—N2—C5—C4	0.3 (5)	C2—C1—C11—C14	135.0 (4)
C3—N2—C5—C6	−179.1 (3)	C4—C1—C11—C14	−47.6 (5)
C1—C4—C5—N2	−0.2 (5)	C2—C1—C11—C13	−106.1 (4)
C1—C4—C5—C6	179.2 (3)	C4—C1—C11—C13	71.3 (4)
C8—N1—C6—C7	−0.6 (4)	C2—C1—C11—C12	11.1 (5)
C8—N1—C6—C5	179.2 (3)	C4—C1—C11—C12	−171.5 (4)
N2—C5—C6—N1	−19.3 (4)	C7—C10—C15—C16	−56.6 (4)
C4—C5—C6—N1	161.3 (3)	C9—C10—C15—C16	124.1 (4)
N2—C5—C6—C7	160.5 (3)	C7—C10—C15—C18	−178.2 (3)
C4—C5—C6—C7	−19.0 (5)	C9—C10—C15—C18	2.5 (5)
N1—C6—C7—C10	−0.1 (4)	C7—C10—C15—C17	62.0 (4)
C5—C6—C7—C10	−179.8 (3)	C9—C10—C15—C17	−117.3 (4)

### Hydrogen-bond geometry (Å, °)

**Table:**

*D*—H···*A*	*D*—H	H···*A*	*D*···*A*	*D*—H···*A*
N1—H1N···O1^i^	1.00 (3)	1.89 (3)	2.716 (4)	137 (3)
C4—H4···O3^ii^	0.93	2.58	3.480 (4)	164
C7—H7···O3^ii^	0.93	2.49	3.389 (4)	163
C9—H9···O3^iii^	0.93	2.60	3.385 (4)	143

Symmetry codes: (i) −*x*+1, −*y*+1, *z*−1/2; (ii) *x*−1/2, −*y*+1/2, *z*; (iii) −*x*+1/2, *y*−1/2, *z*−1/2.

## References

[bb1] Bruker (2002). *SMART* and *SAINT* Bruker AXS Inc., Madison, Wisconsin, USA.

[bb2] Bruker (2005). *SADABS* Bruker AXS, Inc., Madison, Wisconsin, USA.

[bb3] Duan, L. L., Xu, Y. H., Zhang, P., Wang, M. & Sun, L. C. (2010). *Inorg. Chem.* **49**, 209–215. 10.1021/ic9017486 19994841

[bb4] Farrugia, L. J. (1997). *J. Appl. Cryst.* **30**, 565.

[bb5] Farrugia, L. J. (1999). *J. Appl. Cryst.* **32**, 837–838.

[bb6] Huertas, S., Hissler, M., McGarrah, J. E., Lachicotte, R. J. & Eisenberg, R. (2001). *Inorg. Chem.* **40**, 1183–1188. 10.1021/ic001018d 11300816

[bb7] Morrow, J. R. & Trogler, W. C. (1989). *Inorg. Chem.* **28**, 1330–2333.

[bb8] Noro, S., Kitagawa, S., Kondo, M. & Seki, K. (2000). *Angew. Chem. Int. Ed.* **39**, 2081–2084. 10.1002/1521-3773(20000616)39:12<2081::aid-anie2081>3.0.co;2-a 10941021

[bb9] Qin, Z. Q., Jennings, M. C. & Puddephatt, R. J. (2002). *Inorg. Chem.* **41**, 3967–3974. 10.1021/ic020227q 12132923

[bb10] Sheldrick, G. M. (2008). *Acta Cryst.* A**64**, 112–122. 10.1107/S0108767307043930 18156677

[bb11] Yaghi, O. M., Li, H., Davis, C., Richardson, D. & Groy, T. L. (1998). *Acc. Chem. Res.* **31**, 474–484.

